# Supplementary material for: Source Identification and Genome-Wide Association Analysis of Crown Rot Resistance in Wheat
Source: Plants (Basel). 2022 Jul 24;11(15):1912. doi: 10.3390/plants11151912 (PMC9329777; doi:10.3390/plants11151912)
Supplement: Supplementary file 1 [file plants-11-01912-s001.zip › Table S1.pdf]

Table S1. Excellent varieties/lines resistant to CR in two years.

years	Varieties	DI	Resistance	Types	W&S	Sources
2019	Fielder	5.6	R	cultivar	S	the United States
	Chaodasui	6.7	R	cultivar	I	China
	Zimai12	6.7	R	cultivar	I	Huang-huai Winter Wheat Region
	Huximai	10.8	R	landrace	I	Winter Wheat Region of Middle and Lower Yangtze River
	SIRMIONE	11.7	R	cultivar	W	Italy
	Xinong509	11.8	R	cultivar	I	Huang-huai Winter Wheat Region
	Hyden	12.5	R	cultivar	S	Australia
	Sifangmai	13	R	landrace	S	Southwestern Winter Wheat Region
	Dawson	13	R	cultivar	W	Canada
	Ji954072	13	R	breeding line	W	Huang-huai Winter Wheat Region
	Mazhamai	13.5	R	landrace	I	Huang-huai Winter Wheat Region
	Zhengmai9405	14.9	R	cultivar	I	Huang-huai Winter Wheat Region
	Xuke718	15	R	cultivar	I	Huang-huai Winter Wheat Region
	Yuqiumai	15.2	MR	landrace	I	Southwestern Winter Wheat Region
	OPATA	15.2	MR	cultivar	S	Mexico
	Xibei612	15.2	MR	cultivar	W	Huang-huai Winter Wheat Region
	Liaomai16	15.5	MR	cultivar	I	Huang-huai Winter Wheat Region
	Owens	16.7	MR	cultivar	S	the United States
	Jimai38xinxi	17.1	MR	cultivar	I	Northern Winter Wheat Region
	Luomai26	18.1	MR	cultivar	I	Huang-huai Winter Wheat Region
	Aguilal	18.2	MR	cultivar	S	Morocco
	Zhongmai895	18.3	MR	cultivar	I	Northern Winter Wheat Region
	Heng6632	18.5	MR	cultivar	I	Huang-huai Winter Wheat Region
	Baihuamai	18.6	MR	landrace	S	Southwestern Winter Wheat Region
	Ron 2-Fnd×CMH74A.630	18.6	MR	breeding line	S	Mexico
	Yumai13	19	MR	cultivar	S	Southwestern Winter Wheat Region
	Mianyang26	19.2	MR	cultivar	S	Southwestern Winter Wheat Region
	Lantian31	19.2	MR	cultivar	W	Northwestern Spring Wheat Region

	Zhenmai168	19.4	MR	cultivar	S	Winter Wheat Region of Middle and Lower Yangtze River
	VAIOLET	19.8	MR	cultivar	NA	the United States
	Soissons	20	MR	cultivar	W	France
	Zhongyu12	20.1	MR	cultivar	I	Huang-huai Winter Wheat Region
	Shuiyuan86	20.4	MR	cultivar	W	Korea
	Boai7023	20.4	MR	cultivar	S	Huang-huai Winter Wheat Region
	Zhongmai99	22.1	MR	breeding line	I	Huang-huai Winter Wheat Region
	Jichun1016	22.5	MR	cultivar	S	Northeastern Spring Wheat Region
2020	Youmanghong7	6.3	R	cultivar	W	Northern Winter Wheat Region
	Yanmai8911	9	R	cultivar	I	Huang-huai Winter Wheat Region
	RED EGYPTIAN	10.1	R	landrace	S	Egypt
	Huximai	10.4	R	landrace	I	Winter Wheat Region of Middle and Lower Yangtze River
	GEHEN KANAK	10.4	R	landrace	S	Indonesia
	Mazhamai	11.1	R	landrace	I	Huang-huai Winter Wheat Region
	Yuqiumai	11.5	R	landrace	I	Southwestern Winter Wheat Region
	Sifangmai	12.5	R	landrace	S	Southwestern Winter Wheat Region
	Zhenmai168	12.9	R	cultivar	S	Winter Wheat Region of Middle and Lower Yangtze River
	Xinong509	13.9	R	cultivar	I	Huang-huai Winter Wheat Region
	Dawson	14.2	R	cultivar	W	Canada
	Freedom	14.2	R	cultivar	W	the United States
	Xinyang12	14.2	R	cultivar	S	Huang-huai Winter Wheat Region
	Zimai12	15	R	cultivar	I	Huang-huai Winter Wheat Region
	Yumai70	15.3	MR	cultivar	I	Huang-huai Winter Wheat Region
	Ron 2-Fnd×CMH74A.630	15.8	MR	breeding line	S	Mexico
	Fothand	16.3	MR	NA	NA	NA
	Dahuangpi	16.3	MR	landrace	I	Winter Wheat Region of Middle and Lower Yangtze River
	Aguilal	16.4	MR	cultivar	S	Morocco
	Yunmai34	16.5	MR	cultivar	S	Southwestern Winter Wheat Region
	SIRMIONE	16.7	MR	cultivar	W	Italy
	Mianyang26	16.7	MR	cultivar	S	Southwestern Winter Wheat Region

Ganmai8	16.7	MR	cultivar	S	Northwestern Spring Wheat Region
Humai15-1	16.7	MR	cultivar	S	Winter Wheat Region of Middle and Lower Yangtze River
Luyuan502	16.7	MR	cultivar	I	Huang-huai Winter Wheat Region
Changzhi6406	17.2	MR	cultivar	W	Northern Winter Wheat Region
Chayazheda29	17.3	MR	landrace	S	Qinghai-Tibet Spring and Winter Wheat Region
Xibei612	17.4	MR	cultivar	W	Huang-huai Winter Wheat Region
Zhongyu12	17.4	MR	cultivar	I	Huang-huai Winter Wheat Region
Wanmai19	17.8	MR	cultivar	I	Winter Wheat Region of Middle and Lower Yangtze River
Colt	17.9	MR	cultivar	W	the United States
Owens	18.1	MR	cultivar	S	the United States
VAIOLET	18.1	MR	cultivar	NA	the United States
Jichun1016	18.1	MR	cultivar	S	Northeastern Spring Wheat Region
Gaoyou2018	18.1	MR	cultivar	I	Northern Winter Wheat Region
Liaomai16	18.3	MR	cultivar	I	Huang-huai Winter Wheat Region
Heng6632	18.3	MR	cultivar	I	Huang-huai Winter Wheat Region
RIETI	18.3	MR	landrace	W	Italy
PURPLESTRAW	18.3	MR	cultivar	I	the United States
Sumai3	18.3	MR	cultivar	S	Winter Wheat Region of Middle and Lower Yangtze River
Xiaoyan81	18.8	MR	cultivar	I	Huang-huai Winter Wheat Region
Bohemia	19.4	MR	cultivar	W	Czech
Zhengmai9405	19.4	MR	cultivar	I	Huang-huai Winter Wheat Region
Linmai4	19.4	MR	cultivar	I	Northwestern Spring Wheat Region
Pusa 6	19.6	MR	landrace	W	Indonesia
Suneca	19.6	MR	cultivar	S	Australia
Jinmai30	19.7	MR	cultivar	I	Northern Winter Wheat Region
Yannong19	19.7	MR	cultivar	W	Huang-huai Winter Wheat Region
OPATA	20	MR	cultivar	S	Mexico
Huaimai25	20	MR	cultivar	I	Winter Wheat Region of Middle and Lower Yangtze River
Vicam F70	20	MR	breeding line	S	Mexico
Yumai15	20.1	MR	cultivar	I	Huang-huai Winter Wheat Region

Luomai26	20.4	MR	cultivar	I	Huang-huai Winter Wheat Region

S represents that the variety is a spring variety/line, W is a winter variety/line, I is a semi-winter variety/line, and NA is missing information.
