# Supplementary material for: Source Identification and Genome-Wide Association Analysis of Crown Rot Resistance in Wheat
Source: Plants (Basel). 2022 Jul 24;11(15):1912. doi: 10.3390/plants11151912 (PMC9329777; doi:10.3390/plants11151912)
Supplement: Supplementary file 1 [file plants-11-01912-s001.zip › Table S2.pdf]

Table S2. Classification of sub groups of varieties/lines in the population.

Sub groups	Varieties/lines
Group 1 (Cyan)	Huximai, Mazhamai, Yuqiumai, Sifangmai, Dahuangpi, Zipi, Mangmai, Shuiyuan86, Glenlea, Wuxumai, Huangguaxian, Pingyuan50, Ningmai9, Shannong205, Bima4, Huoliaomai, Jiangdongmen, Hanxuan10, HINDI, Hongkeyoumang, Jinfeng1, Liuzhutou, Dingxingzhai, Jiyumai, Yaanzao, Sanyuehuang, Xifeng, Fumai, Gezhoumai, Meiqianwu, Xiaofoshou, Louguding, Mingxian169, Hogmai, Tuotuomai, Hongheshangtou, Kangdingxiaomai, Baimaizi, Shanmai, Hongxiuzi, Youzimai, Zhemai1, Baihuamai, Baiyoumai, Weimai4, Baipu, Nongda183, Jingyang60, Hongmangyouzimai, Tumangmai
Group 2 (purple)	Youmanghong7, Yanmai8911, Xibei612, Jinmai30, OPATA, Yumai15, Guixie3, AimengniuV, Dongfanghong3, Jinan16, Xuzhou8, Xiannong39, Maohuiafu, Lumai5, Pubing202, Flanders, Lumai11, Xinmai16, Een1, Yanzhan1, Hezuo4, Chaodasui, Nongda36, Baonong8865-15-25, Ningmai13, Xingzi9104, Xinong1376, Maoyingafu, ZImai, Jingnong79-13, Xinong3517, Lumai3, Lumai12, Xiaoyan4, Xinong889, Shannong1, Xindong20, Jinan8, Zhengzhou17, Kenong9204, Changwu134, Mainong791, Shijiazhuang8, AimengniuIV, Wanmai50, Lanli, Yumai18, Yumai19, Shannong9, Baihuomai, Jinan4, Xinong979
Group 3 (orange)	Xinyang12, Zimai12, Ailiduo, Chayazheda29, Zhingyu12, Jichun1016, Xiaoyan81, Luomai26, Neixiang182, Zhoumai16, Taishan4, Colotana, Jinmai54, Taixue12, Zhengmai9023, Gaoyou503, Zhoumai22, Xingmai8, Bainong3217, Shan229, Shannong12, Jingzhou66, Ji5265, Nanda2419, Xinmai9, Shimai18, Ourou, Jiaomai266, Huamai5, Taikong6, Zhoumai12, Ruihuamai518, Shiluan02-1, Guomai301, Zhouluo08-2, Yumai54, Ruihuamai518, Shanyou225, Xinmai26, Jingmai79, Aikang58, FILIN, Haocheng9411（c02667）, Xinfumai1, Abo, Xingmai1, Zhou8425B, Huaichuan916, Aifeng3, Shanmai159, Yumai2, Sumai188, Xiaoheimai, SANGIACOMO, Xuke718, Luyuan301, Kaimai21, Yumai21(Zhoumai9), Jining13, Zhongyu9398, Jimai24, Paozimai, Anke0817, Xinong88, Zhongxin78, Xunong7, Kenong199, Chuanmai36, Shi-4185, Longping203, Ningchun13, Zhongluo08-1
Group 4 (yellow)	Xinong509, Luyuan502, Gaoyou2018, Yannong19, Huaimai25, Xiangmai969, Huaimai18, Changmai6135, Jimai45, Xinong364, Ji954072, Yikemai1506, Zhongmai99, Zhengmai151, Luomai18, Pingan8, Guomai0319, Chang6359, Yumai58, Lumai14, 935106, Luomai906, Rumai0319, Yannong15, Xuke1, Yannong5851, Jimai43, Lumai21, Su533, Jimai60, Ruihua549, Shanmai139, Shannong14, Yannong0428, Baiyoubao, Fanmai8, Zhengmai7598, Luomai4, Jimai229, Jimai20, Hemai17, Zhongmai578, Jimai19, Qingchun5, Pingan6, Jimai44, Huaimai22, Zhongyu10, Yumai47, Shannong116, Shannong15, Wanmai68, Weimai6, Qingnong2, Xinong658, Huaimai20, Luomai21, Yannong999, Huaimai1196, Shannong20, Weimai4046, Pumai9, Heimai1, Tainong18, Xuyan5, Huaimai304, Zhengmai1860, Luomai22, Xinong528, Zhoumai36, Xiangmai25, Wanfeng269, Xinong9871, Jimai20, Jinan17, Zhongnongmai4008, Hemai1, Wenmai6, Zhengnong17, Zhongmai895, Ruihuamai520

Group 5 (green)	Dawson, Fothand, Aguilal, Yunmai34, Colt, Heng6632, Bohemia, Zhengmai9405, Suneca, Vicam F70, OPATA/RAYON//KAUZ/3/PFAU/MILAN, Lantian31, Jinmai47, Kosutka, Hanyou504, Yaomai16, Lantian26, FLORKWA-2/6/SAKER´S´/5/RBS/ANZA, MUNIA//CHEN/ALTAR 84/3/CHEN/AEGILOPS SQUARROSA(TAUS)//BCN/4/MARCHOUCH-8, Siete Cerros 66, Yunmai56, Changhan58, Luohan1, Jinmai90, Banquet, Jimai32, Reeves, Liangxing99, Secese, Wilhelmina, SAFI-1/ZEMAMRA-1, LinY8159, ALTTAR 84/AE.SQUARROSA(219)//SERI/3/MASSIRA, Magong, Xinong928, Yangguang851, Yumai48, Hartog, Bodycek, Yannong836, Lanhangxuan121, Gabo, HUBARA-5/ANGI-1, Shannong45, Fronteira, Jimai3, Lanhangxuan122, Yunhan20410, Jinmai31, KARAWAN-1/TALLO 3//JADIDA-2, Pavon76, Arduini, TINAMOU-2//TEVEE-1/SHUHA-6, Luohan11, MOUKA-4*2/4/KEA´S´/3/MN72252//HD2170/BOW´S´, Luohan13, MELLAL-1/OUEDZEM-1, Changwu521, SERI.1B*2/3/KAUZ*2/BOW//KAUZ/4/KAUZ/FLORKWA-1, Heng136, Matylda, Yunhan618, ZEMAMRA-5/ZEMAMRA-5, Xinong1043, Heshengerhao, Heng4399, Luohan9, Buc/Bjy, Jing771, Lusitano (S), Hyden, Luohan2, Yunhan719, Bodallin, Hengguan35, KAUZ´S´/FLORKWA-1//GOUMRIA-3, Caoxuan5, Pitic62, Liangxing77, Dan5093, Shannong253, Changmai251, Penjamo 62, MASSIRA/SAFI-1, Yunhan22-33, Yunhan805, Yujiao5, Jinghe90jian15, Linkang11, Chang6878, Ciano, Saada, Seladon, LermaRojo64
Group 6 (red)	Zhenmai168, Yumai70, Mianyang26, Ganmai8, Wanmai19, VAIOLET, Sumai3, Pusa 6, Ningmai15, Yumai13, Chuanmai22, Mianyang31, Chanmai104, Emai11, Sumai3, Zhengyin1, Ningnuomai1, Chuanyu6, Wuyimai, CENTAURD, 92R137 , Mianyang20, Shuwan8, Emai16, Fan6, Yangmai18, Yangmai16, Neimai11, Neijiang31, Xikemai6, Jinmai2148, Emai6, Mianmai37, Yangmai1, Yumai13, Sumai6, Yanshi4, Zhongjiao2, Chuanmai8, SALGEMMA, Baixiaomai, Neixiang185, Neimai836, SAGITTARIO, Ningmai3, Chuanmai107, Knteh, Xiannong151, Xikemai2, Xiaoyan22, Zhengzhou4, Zhenmai6, Emai580, Exi84-1031, Chuanmai42, Longchun8, Wanmai33, Xian8, Xuzhou21, Gaoyuan602, Mianyang11, Becejka, Yangmai158, Boai7023, Chixiaomai
Group 7 (light blue)	RED EGYPTIAN, GEHEN KANAK, ROMANIAN, Ron 2-Fnd×CMH74A.630, SIRMIONE, Owens, PURPLESTRAW, Baletka, Napo 63, ABYSSINIAN 43, Indexa, Viginta, Camacrania, Dagmar, RED KAMSERETI, Hamedan 1, AC Foremost, KHOMROK, MACS-WHITE, ENESCO, JOHN-BROWN, EARLY BLACKHULL, Penawa, SUNSET, AUSTRAL, SALTI NAIMYA, SENMARQ, SARSABAZ, Dromedaris, PENNY, RASH KOOL, ROOI SPITSKOP, Gaboto, FARO, Amazon, Banks, GLUYAS EARLY, Sunelg, Parsee, LAGEADINHO, AC vista, Hungarian Dm 15, CHIH95.10.1, Garent, Krac 66, BECFIORE, AURORE 4323 A, IRAGI, Fielder, O.S. Jakowski, Duiker, Jacui, Tukaminia, PRIMOASI, PADUS, AC Nanda, Saet, Mexifen, GHABAGHEB, Plainsman C.I.14128, REDFIFE, FIFE, HOMARA NO 44, Avocet, IWA 8606268, MISHRAGANI, Fln-Acc×Ana 75

Group 8 (dark blue)	Freedom, Changzhi6406, Liaomai16, RIETI, Linmai4, Jimai38xinxi, Jingdong22, SERIO, Xiaobaimang, Jinghua2, Fengkang8, Heibao, Zhengzhou9860, Anhui11, Qianjiaomai, Shimai15, Dan6172, Pingyang181, Zhongmai175, Jinan9, Yannong22, Shijiazhuang54, Zhengmai9201, Dan4565, Songhuajiang1, Tangmai2, Beijing10, Jimai6, Linmai2, Zhongmai9, Taishan1, Jinan13, Lumai7, Beijing8, Shanqianmai, Jimai1, Taishan23, Shiyou20, Zaoyangmai, Attila, Fenghan3, Xuzhou24, Qingfeng1, Huabei187, Jingshuang16, Taishan21, Kehong1, Nongda211, Jimai26, Zhongyou9507, Xinchun2, Wanyuan-66, Laizhou953, Changfeng4, Hanxuan3, Zhongmai415, Lunxuan987, Xinke9, Kanghuixianhong, Beijing0045, Jinan2, Luomai23, Jing411, Zhongyou206, Huabei187, Wennongliuhao, Soissons
